# Clinical practice patterns in uveitic cataract surgery: a national survey

**DOI:** 10.1186/s12886-026-05328-0

**Published:** 2026-09-10

**Authors:** Maham Imran, Muhammad Imran Saleem Channar, Sarmad Imran, Maryum Khilji, Bijay Tamang, Abdulhadi M. A. Mahgoub, Mohammedsadeq A. Shweliya

**Affiliations:** 1 Bahawal Victoria Hospital, Bahawalpur, Pakistan; 2 Quaid-e-Azam Medical College, Bahawalpur, Pakistan; 3 Khyber Teaching Hospital, Peshawar, Pakistan; 4 Faculty of Medicine, University of Gezira, Dongola, North Sudan, 2111 Sudan; 5 University of Baghdad College of Medicine, Baghdad, Iraq

**Keywords:** Uveitic cataract, Perioperative management, Practice patterns, National survey, Pakistan

## Abstract

**Background:**

Cataract is a frequent and visually significant complication of uveitis, yet perioperative management practices vary widely and are not always guided by standardized protocols, particularly in settings such as Pakistan where uveitis is relatively common. This study aimed to describe current perioperative management practices for uveitic cataract among ophthalmologists in Pakistan, providing an observational baseline to inform future evidence-based consensus rather than prescribing clinical recommendations, and to explore variations based on clinician experience and case volume.

**Methods:**

This descriptive cross-sectional survey included fellowship-trained ophthalmologists in Pakistan actively managing uveitic cataract. A structured, expert-validated questionnaire assessed perioperative inflammation control, intraoperative strategies, postoperative regimens, clinical decision-making, and attitudes toward national guidelines. The survey was administered electronically from December 2025 to February 2026. Responses were stratified by clinical experience and annual case volume, and statistical analysis was performed using chi-square and Fisher’s exact tests.

**Results:**

Fifty-one ophthalmologists responded (51% response rate); 68.6% had ≤ 10 years of post-fellowship experience. A quiescent period of ≥ 3 months was the most common determinant of surgical timing (72.5%). Preoperatively, intensified topical corticosteroids were widely used (72.5%), and nearly half prescribed short systemic steroids (49.0%). Combined pharmacologic–mechanical strategies were preferred intraoperatively (74.5%), and 80.4% routinely implanted a primary IOL when capsular support was adequate, most commonly hydrophobic acrylic (49.0%). Postoperatively, 62.7% used systemic steroids routinely in all cases, and 47.1% routinely prescribed topical NSAIDs for cystoid macular edema prophylaxis. Preoperative macular integrity on OCT was most often identified as the strongest predictor of visual gain (45.1%). Significant differences in steroid intensification and NSAID use were observed according to surgeon experience (*p* < 0.05). Overall, 84.3% supported development of national standardized guidelines.

**Conclusion:**

Among surveyed Pakistani ophthalmologists, significant variability exists in perioperative management of uveitic cataract surgery, influenced by surgeon experience and annual case volume. These findings offer a regional baseline; standardized national guidelines, informed by broader consensus-building, may improve consistency and outcomes in this complex patient population.

## Introduction

Uveitis is a significant cause of visual impairment globally. In South Asia, including Pakistan, it constitutes 10–15% of the patients visiting the tertiary eye care facilities, and the proportion of infectious aetiology is higher than in the Western countries [1]. Cataract is a major complication in patients with long-term history of uveitis, occurring in approximately 40–60% of affected eyes, more often in younger patients, thus increasing socioeconomic burden [2]. Moreover, it remains the major indication of surgical intervention in uveitic eyes. The pathogenesis of uveitic cataract is multifactorial, involving direct inflammatory insult to the crystalline lens epithelium, disruption of the blood-aqueous barrier, posterior synechiae formation with pupillary dysfunction, chronic corticosteroid therapy and the duration of intraocular inflammation [3, 4].

Cataract surgery in uveitic eyes is quite challenging. Preoperatively, eyes often demonstrate posterior synechiae, fibrinous membranes, weak zonules, irregular anterior chamber depth, which can hinder surgical access, making it difficult to achieve adequate mydriasis [5]. Intraoperatively, such cases often necessitate the use of specialized tools and techniques including pupil expanders, modified capsulorrhexis method, which further increases the risk of complications like iris trauma and posterior capsule rupture. The postoperative period is associated with inflammation leading to cystoid macular edema (12% incidence) 6], glaucoma, posterior capsular opacification and other risks. However, recent studies show significant improvement in uveitic cataract surgery visual outcomes due to better perioperative inflammation control, IOL materials and microsurgical techniques [7].

The evidence base for managing uveitic cataract is limited, resulting in treatment decisions, including preoperative and postoperative steroid protocols and intraocular lens implantation approaches, being largely individualized rather than guided by standardized guidelines. A 2021 UK study showed decent outcomes but noted differences in complication rates across centers, emphasizing the need for standardized approaches [8]. Guidelines based on evidence have been encouraged by the American Academy of Ophthalmology (AAO) and Standardization of Uveitis Nomenclature (SUN) Working Group [9, 10].

Pakistan’s ophthalmology landscape is distinct, with uveitis frequently linked to infectious causes [11, 12]. Healthcare resources and specialist expertise are not evenly distributed across the country, which can influence how uveitic cataract is managed in different clinical settings. To our knowledge, no prior study has examined Pakistani ophthalmologists practice patterns in uveitic cataract surgery. This national survey therefore aims to describe current practice patterns, highlight areas of variation or gaps in care, and evaluate clinicians’ views on the need for standardized guidelines. The findings may help inform the development of practical, context-specific strategies to improve the management of uveitic cataract in Pakistan.

## Materials and methods

This descriptive cross-sectional study was conducted among ophthalmologists in Pakistan experienced in managing uveitic cataract to assess real-world perioperative management practices. The target population comprised certified, fellowship-trained ophthalmologists practicing in Pakistan who were actively involved in the perioperative management of uveitic cataract, including uveitis/anterior segment subspecialists and comprehensive ophthalmologists with relevant fellowship training. Ophthalmology trainees (residents/fellows in training) and clinicians not currently managing such cases were excluded.

Since no centralized national registry of uveitis-specialized ophthalmologists exists in Pakistan, a sampling frame was built using a combined purposive and snowball sampling approach. Eligible consultants were identified through the membership directories of professional ophthalmology societies, including the Pakistan Ophthalmological Society and relevant subspecialty groups. Additional consultants were identified through institutional email lists of major tertiary care and teaching hospitals across the country, as well as verified professional social media/networking groups (e.g., WhatsApp and Facebook groups restricted to practicing ophthalmologists). This process yielded an initial list of 100 identifiable, eligible consultants who were formally invited to participate. Of these, 51 completed the survey, giving a response rate of 51% (51/100).

No centralized national registry documents the exact number of fellowship-trained ophthalmologists in Pakistan who actively manage uveitic cataract. This represents a narrow subspecialty overlap between uveitis/anterior segment training and routine surgical practice. Our sampling frame included 100 identifiable and eligible consultants. These consultants were identified through professional society directories, institutional networks, and verified professional groups. This sampling frame was believed to capture a substantial proportion of ophthalmologists in Pakistan who were actively practicing in this specific area, given the relatively small and interconnected nature of this subspecialty community nationally. The achieved sample of 51 respondents demonstrated meaningful engagement from this specialized clinical group. This supported the reasonable generalizability of the findings to national practice patterns. Nonetheless, as with any non-registry-based sampling approach, complete population coverage could not be confirmed. Isolated practitioners without society affiliation or institutional networking may have been underrepresented.

A structured questionnaire was developed after reviewing international guidelines, including those from the American Academy of Ophthalmology (AAO) and the Standardization of Uveitis Nomenclature (SUN) Working Group, along with relevant published literature [9, 10]. The questionnaire was reviewed by senior uveitis and anterior segment specialists to ensure its relevance, clarity, and clinical applicability. It consisted mainly of multiple-choice questions and included questions about demographics, control of preoperative inflammation, preferences of surgical technique and type of intraocular lens (IOL), postoperative management, clinical scenarios and opinions regarding the need of a national guideline.

The analysis included both descriptive and inferential statistics. All variables in the study were categorical so frequencies and percentages were calculated and tabulated. To assess association among categorical variables, Chi square test and Fischer Exact test (where the expected count in cells was less than 5) was used. Statistical significance was established at a p-value of 0.05 or less with a 95% confidence interval. All statistical calculations were performed using SPSS Software (IBM, version 27.0.1).

## Results

There were 51 ophthalmologists who responded to the survey. Most respondents had ≤ 10 years of post-fellowship experience (68.6%), while 31.4% had more than 10 years. The majority practiced in teaching or tertiary care institutions (66.7%), with the rest working in mixed or non-teaching settings. Most participants were based in Punjab (66.7%). Regarding cataract surgery volume, 68.6% of respondents managed ≤ 25 uveitic cataract cases per year, while 31.4% reported managing > 25 cases annually (Table 1).

**Table 1:** Practice Characteristics and Clinical Experience of participating ophthalmologists (*n* = 51)

		N (%)
Post-Fellowship Practice Duration (Clinical experience following primary fellowship qualification: FCPS, MS, FRCS, FRCOphth)	3–5 years	22 (43.1%)
	6–10 years	13 (25.5%)
	11–15 years	11 (21.6%)
	> 15 years	5 (9.8%)
Practice Setting	Mixed Practice	10 (19.6%)
	Primary/Secondary care public sector hospital	2 (3.9%)
	Private Setup	2 (3.9%)
	Teaching/ Tertiary Care Institution	34 (66.7%)
	Trust Hospital/ NGO	3 (5.9%)
Province/Region of Practice	Balochistan	3 (5.9%)
	Islamabad/ Gilgit Baltistan/ AJK	2 (3.9%)
	Khyber Pakhtunkhwa	6 (11.8%)
	Punjab	34 (66.7%)
	Sindh	6 (11.8%)
Approximate number of uveitic cataract surgeries per Annum	< 10	18 (35.3%)
	10–25	17 (33.3%)
	26–50	5 (9.8%)
	50–100	6 (11.8%)
	>100	5 (9.8%)

N: Frequency, %: Percentage, FCPS: Fellowship of the College of Physicians and Surgeons, MS: Master of Surgery, FRCS: Fellow of the Royal College of Surgeons, FRCOphth: Fellow of the Royal College of Ophthalmologists, NGO: Nongovernmental organization, AJK: Azad Jammu and Kashmir

Most respondents reported a fixed inflammation-free period of at least 3 months as the primary determinant for timing of uveitic cataract surgery (72.5%). Preoperatively, intensified topical corticosteroids were the most commonly employed strategy for inflammation control (72.5%), while nearly half of the surgeons also used a short course of systemic steroids (49.0%). In cases of infectious uveitis, the majority preferred to defer surgery until quiescence without antimicrobial coverage (56.9%). Intraoperatively, a combined pharmacologic and mechanical approach was the preferred strategy for managing miosis or posterior synechiae (74.5%). Most surgeons routinely implanted a primary intraocular lens when adequate capsular support was present (80.4%), with hydrophobic acrylic lenses being the most commonly preferred IOL material (49.0%). Subconjunctival steroid administration was the most frequently used intraoperative anti-inflammatory adjunct (43.1%) (Table 2).

**Table 2:** Pre- and intraoperative management of uveitic cataract surgery

Question / Item	Response	*N* (%)
**PREOPERATIVE MANAGEMENT**		
Primary determinant of surgical timing¹	Degree of inflammatory control on current regimen	18 (35.3%)
	Patient’s visual or functional demand despite low-grade inflammation	16 (31.4%)
	Fixed duration of quiescence (≥ 3 months)	37 (72.5%)
	Disease subtype–specific considerations (e.g., JIA, VKH, Behçet)	3 (5.9%)
Preoperative inflammation control measures¹	Intensified topical corticosteroids (1–2 hourly for 1–2 weeks)	37 (72.5%)
	Short course of systemic steroids	25 (49.0%)
	Periocular or intravitreal steroid	14 (27.5%)
	Optimization of maintenance immunosuppression/biologic therapy	11 (21.6%)
Perioperative antimicrobial strategy in infectious uveitis	Continue existing antimicrobial therapy uninterrupted through surgery	7 (13.7%)
	Defer surgery until quiescence without additional antimicrobial coverage	29 (56.9%)
	Initiate new, targeted antimicrobial prophylaxis preoperatively	8 (15.7%)
	No routine antimicrobial prophylaxis	7 (13.7%)
Perioperative management of patients on systemic immunosuppression/biologic therapy	Add perioperative systemic corticosteroid cover	11 (21.6%)
	Continue the current regimen without modification	17 (33.3%)
	Defer regimen changes and timing to the managing rheumatologist/internist	2 (3.9%)
	Individualized decision based on drug, dose, and inflammation status	20 (39.2%)
	Withhold the biologic/immunosuppressive dose in the immediate perioperative period	1 (2.0%)
Timing of preoperative topical corticosteroid intensification	1 week before surgery	16 (31.4%)
	1–3 days before surgery	10 (19.6%)
	2–4 weeks before surgery	8 (15.7%)
	Only if active inflammation present	17 (33.3%)
**INTRAOPERATIVE CONSIDERATIONS**		
Management of intraoperative miosis/posterior synechiae	Combined pharmacologic and mechanical approach	38 (74.5%)
	Mechanical synechiolysis	4 (7.8%)
	Pharmacologic synechiolysis (viscoelastic)	2 (3.9%)
	Pupil expansion device (e.g., Iris hooks/Malyugin ring)	7 (13.7%)
Approach to intraocular lens (IOL) implantation	Avoid primary IOL implantation only in special situations (e.g., in children/JIA, or high-risk cases)	5 (9.8%)
	Implant only if inflammation had been quiescent for a defined pre-op period (e.g., ≥ 3 months)	5 (9.8%)
	Routine primary IOL implantation when capsular support adequate	41 (80.4%)
Preferred IOL material	Heparin-surface-modified (HSM) PMMA	20 (39.2%)
	Hydrophilic acrylic	6 (11.8%)
	Hydrophobic acrylic	25 (49.0%)
Preferred intraoperative anti-inflammatory adjunct	Intracameral steroid (Prednisolone/Dexamethasone)	11 (21.6%)
	Intravitreal Triamcinolone	1 (2.0%)
	No adjunct (standard postoperative regimen only)	6 (11.8%)
	Posterior Sub-Tenon’s Triamcinolone	11 (21.6%)
	Subconjunctival steroid (Prednisolone/Dexamethasone)	22 (43.1%)

N: Frequency, %: Percentage, ^1^Multiple responses permitted, JIA: Juvenile idiopathic arthritis, VKH: Vogt Koyanagi Harada disease

Postoperatively, most respondents routinely prescribed systemic corticosteroids for uveitic cataract patients (62.7%), while 13.7% reserved their use for high-risk cases. The majority of surgeons tailored the duration of topical corticosteroid therapy according to inflammation response and uveitis type (60.8%). Nearly half of respondents routinely prescribed topical NSAIDs for cystoid macular edema prophylaxis (47.1%), whereas 31.4% initiated NSAIDs only after CME detection. For postoperative management, mydriatic-cycloplegic therapy was either prescribed routinely (43.1%) or selectively based on anterior chamber activity (45.1%), and follow-up schedules were most commonly individualized according to disease activity (47.1%). In cases of inflammation refractory to maximal topical therapy, systemic corticosteroids (41.2%) and sub-Tenon’s steroid injections (37.3%) were the preferred first-line interventions. Persistent subclinical uveitis was the most frequently suspected cause of postoperative CME (56.9%), while preoperative macular integrity on optical coherence tomography (OCT) was considered the strongest predictor of postoperative visual gain (45.1%). A strong majority of respondents supported the establishment of national standardized management guidelines for uveitic cataract surgery (84.3%) (Table 3).

**Table 3:** Postoperative management and clinical decision-making preferences

Question / Item	Response	*N* (%)
**POSTOPERATIVE REGIMEN**		
Postoperative systemic corticosteroid strategy	Avoid routine use; reserved for inflammatory surge	12 (23.5%)
	Routine use in all uveitic cataract patients	32 (62.7%)
	Routine use in high-risk cases only	7 (13.7%)
Duration of postoperative topical corticosteroid therapy	3 months	3 (5.9%)
	4 weeks	7 (13.7%)
	6–8 weeks	10 (19.6%)
	Tailored to inflammation response and uveitis type	31 (60.8%)
Approach to use of post-op topical NSAIDs for CME prophylaxis?	Avoid; rely on topical corticosteroids alone for prophylaxis	6 (11.8%)
	Initiate only if CME is detected postoperatively (OCT/angiography)	16 (31.4%)
	Prescribe only for high-risk cases (e.g., posterior uveitis, prior CME)	5 (9.8%)
	Routine use in all cases	24 (47.1%)
Use of postoperative mydriatic–cycloplegic therapy	Not routinely prescribed	6 (11.8%)
	Prescribe only if anterior chamber activity or synechiae risk is observed	23 (45.1%)
	Routine use for 1–2 weeks in all cases	22 (43.1%)
Postoperative follow-up protocol	Intensive protocol	17 (33.3%)
	Standard cataract surgery protocol	10 (19.6%)
	Tailored based on disease activity	24 (47.1%)
**PRACTICAL MANAGEMENT DECISIONS**		
First-line intervention for inflammation refractory to maximal topical therapy	Sub-Tenon’s steroid injection	19 (37.3%)
	Initiate/augment systemic corticosteroids	21 (41.2%)
	Intensify topical steroid frequency	1 (2.0%)
	Investigate for infection or retained lens material	10 (19.6%)
Primary suspected cause of postoperative CME with quiet anterior chamber	Breakdown of the blood-aqueous barrier	15 (29.4%)
	IOL-related biocompatibility issue	2 (3.9%)
	Overly rapid tapering of topical steroids	5 (9.8%)
	Persistent subclinical uveitis	29 (56.9%)
Strongest predictor of postoperative visual gain	Anatomical type of uveitis	13 (25.5%)
	Patient’s anticipated compliance with therapy	2 (3.9%)
	Preoperative duration of uveitis quiescence	13 (25.5%)
	Preoperative macular integrity on OCT	23 (45.1%)
Support for national standardized management guidelines for uveitic cataract surgery?	Cautiously support; dependent on data confidentiality and utility	8 (15.7%)
	Strongly support; essential for standardizing care	43 (84.3%)

N: Frequency, %: Percentage

When stratified by post-fellowship clinical experience, several significant differences in perioperative management preferences were observed. Surgeons with ≤ 10 years of experience were significantly more likely to intensify topical corticosteroids 1–2 hourly for 1–2 weeks preoperatively as part of preoperative inflammation control compared with those having > 10 years of experience (82.9% vs. 50.0%; *p* = 0.021). Moreover, those with more than 10 years of experience added short systemic steroids more frequently as compared to those with less than 10 years of experience (68.8% vs. 40.0%). A significant association was also identified in postoperative NSAID use for cystoid macular edema (CME) prophylaxis. More experienced surgeons (> 10 years) were significantly more likely to routinely prescribe topical NSAIDs in all uveitic cataract cases (75.0% vs. 34.3%), whereas less experienced surgeons more commonly initiated NSAIDs only after CME detection (42.9% vs. 6.3%; *p* = 0.003). Additionally, a borderline difference was observed in the duration of postoperative topical corticosteroid therapy, with surgeons having ≤ 10 years of experience more frequently tailoring steroid duration based on inflammation response and uveitis type, while those with > 10 years of experience more often favored fixed-duration regimens (*p* = 0.052). No other perioperative, intraoperative, or postoperative management preferences showed statistically significant associations with post-fellowship clinical experience (*p* > 0.05) (Table 4).

**Table 4:** Comparison of perioperative management strategies by post-fellowship clinical experience

Question / Item	Response	Post-fellowship experience, N (%)		P value
		≤ 10 years	> 10 years	
Primary determinant of surgical timing	Degree of inflammatory control on current regimen	10 (28.6%)	8 (50.0%)	0.137
	Patient’s visual or functional demand despite low-grade inflammation	9 (25.7%)	7 (43.8%)	0.198
	Fixed duration of quiescence (≥ 3 months)	26 (74.3%)	11 (68.8%)	0.742
	Disease subtype–specific considerations (e.g., JIA, VKH, Behçet)	1 (2.9%)	2 (12.5%)	0.229
Preoperative inflammation control measures	Intensified topical corticosteroids (1–2 hourly for 1–2 weeks)	29 (82.9%)	8 (50.0%)	0.021*
	Short course of systemic steroids	14 (40.0%)	11 (68.8%)	0.057
	Periocular or intravitreal steroid	10 (28.6%)	4 (25.0%)	1.000
	Optimization of maintenance immunosuppression/biologic therapy	8 (22.9%)	3 (18.8%)	1.000
Perioperative management of patients on systemic immunosuppression/biologic therapy	Add perioperative systemic corticosteroid cover	6 (17.1%)	5 (31.3%)	0.712
	Continue the current regimen without modification	12 (34.3%)	5 (31.3%)	
	Defer regimen changes and timing to the managing rheumatologist/internist	1 (2.9%)	1 (6.3%)	
	Individualized decision based on drug, dose, and inflammation status	15 (42.9%)	5 (31.3%)	
	Withhold the biologic/immunosuppressive dose in the immediate perioperative period	1 (2.9%)	0 (0.0%)	
Timing of preoperative topical corticosteroid intensification	1 week before surgery	10 (28.6%)	6 (37.5%)	0.827
	1–3 days before surgery	7 (20.0%)	3 (18.8%)	
	2–4 weeks before surgery	5 (14.3%)	3 (18.8%)	
	Only if active inflammation present	13 (37.1%)	4 (25.0%)	
Approach to intraocular lens (IOL) implantation	Avoid primary IOL implantation only in special situations (e.g., in children/JIA, or high-risk cases)	3 (8.6%)	2 (12.5%)	1.000
	Implant only if inflammation had been quiescent for a defined pre-op period (e.g., ≥ 3 months)	4 (11.4%)	1 (6.3%)	
	Routine primary IOL implantation when capsular support adequate	28 (80.0%)	13 (81.3%)	
Preferred IOL material	Heparin-surface-modified (HSM) PMMA	15 (42.9%)	5 (31.3%)	0.529
	Hydrophilic acrylic	3 (8.6%)	3 (18.8%)	
	Hydrophobic acrylic	17 (48.6%)	8 (50.0%)	
Preferred intraoperative anti-inflammatory adjunct	Intracameral steroid (Prednisolone/Dexamethasone)	7 (20.0%)	4 (25.0%)	0.755
	Intravitreal Triamcinolone	1 (2.9%)	0 (0.0%)	
	No adjunct (standard postoperative regimen only)	5 (14.3%)	1 (6.3%)	
	Posterior Sub-Tenon’s Triamcinolone	6 (17.1%)	5 (31.3%)	
	Subconjunctival steroid (Prednisolone/Dexamethasone)	16 (45.7%)	6 (37.5%)	
Postoperative systemic corticosteroid strategy	Avoid routine use; reserved for inflammatory surge	10 (28.6%)	2 (12.5%)	0.226
	Routine use in all uveitic cataract patients	19 (54.3%)	13 (81.3%)	
	Routine use in high-risk cases only	6 (17.1%)	1 (6.3%)	
Duration of postoperative topical corticosteroid therapy	3 months	0 (0.0%)	3 (18.8%)	0.052
	4 weeks	5 (14.3%)	2 (12.5%)	
	6–8 weeks	6 (17.1%)	4 (25.0%)	
	Tailored to inflammation response and uveitis type	24 (68.6%)	7 (43.8%)	
Approach to use of post-op topical NSAIDs for CME prophylaxis?	Avoid; rely on topical corticosteroids alone for prophylaxis	6 (17.1%)	0 (0.0%)	0.003*
	Initiate only if CME is detected postoperatively (OCT/angiography)	15 (42.9%)	1 (6.3%)	
	Prescribe only for high-risk cases (e.g., posterior uveitis, prior CME)	2 (5.7%)	3 (18.8%)	
	Routine use in all cases	12 (34.3%)	12 (75.0%)	
First-line intervention for inflammation refractory to maximal topical therapy	Sub-Tenon’s steroid injection	14 (40.0%)	5 (31.3%)	0.858
	Initiate/augment systemic corticosteroids	13 (37.1%)	8 (50.0%)	
	Intensify topical steroid frequency	1 (2.9%)	0 (0.0%)	
	Investigate for infection or retained lens material	7 (20.0%)	3 (18.8%)	
Support for national standardized management guidelines for uveitic cataract surgery?	Cautiously support; dependent on data confidentiality and utility	7 (20.0%)	1 (6.3%)	0.409
	Strongly support; essential for standardizing care	28 (80.0%)	15 (93.8%)	

N: Frequency, %: Percentage, *p < 0.05, significant, Chi-square test, Fischer Exact test

When stratified by annual uveitic cataract surgical volume, a significant difference was observed only in the timing of preoperative topical corticosteroid intensification. Surgeons managing > 25 uveitic cataract cases per year were significantly more likely to intensify topical corticosteroids 1 week prior to surgery compared with those managing ≤ 25 cases annually (50.0% vs. 22.9%; *p* = 0.024). In contrast, surgeons with smaller case loads more often started intensified topical corticosteroids 1 to 3 days before surgery, or 2 to 4 weeks before surgery. No statistically significant associations were identified between annual case volume and other variables (Table 5).

**Table 5:** Comparison of perioperative management preferences by annual uveitic cataract surgical volume

Question / Item	Response	Approximate number of uveitic cataract surgeries per Annum, N (%)		P value
		≤ 25 cases	> 25 cases	
Primary determinant of surgical timing	Degree of inflammatory control on current regimen	11 (31.4%)	7 (43.8%)	0.393
	Patient’s visual or functional demand despite low-grade inflammation	13 (37.1%)	3 (18.8%)	0.189
	Fixed duration of quiescence (≥ 3 months)	24 (68.6%)	13 (81.3%)	0.503
	Disease subtype–specific considerations (e.g., JIA, VKH, Behçet)	1 (2.9%)	2 (12.5%)	0.229
Preoperative inflammation control measures	Intensified topical corticosteroids (1–2 hourly for 1–2 weeks)	26 (74.3%)	11 (68.8%)	0.742
	Short course of systemic steroids	18 (51.4%)	7 (43.8%)	0.611
	Periocular or intravitreal steroid	9 (25.7%)	5 (31.3%)	0.742
	Optimization of maintenance immunosuppression/biologic therapy	7 (20.0%)	4 (25.0%)	0.723
Perioperative management of patients on systemic immunosuppression/biologic therapy	Add perioperative systemic corticosteroid cover	9 (25.7%)	2 (12.5%)	0.206
	Continue the current regimen without modification	9 (25.7%)	8 (50.0%)	
	Defer regimen changes and timing to the managing rheumatologist/internist	2 (5.7%)	0 (0.0%)	
	Individualized decision based on drug, dose, and inflammation status	15 (42.9%)	5 (31.3%)	
	Withhold the biologic/immunosuppressive dose in the immediate perioperative period	0 (0.0%)	1 (6.3%)	
Timing of preoperative topical corticosteroid intensification	1 week before surgery	8 (22.9%)	8 (50.0%)	0.024*
	1–3 days before surgery	9 (25.7%)	1 (6.3%)	
	2–4 weeks before surgery	8 (22.9%)	0 (0.0%)	
	Only if active inflammation present	10 (28.6%)	7 (43.8%)	
Approach to intraocular lens (IOL) implantation	Avoid primary IOL implantation only in special situations (e.g., in children/JIA, or high-risk cases)	2 (5.7%)	3 (18.8%)	0.225
	Implant only if inflammation had been quiescent for a defined pre-op period (e.g., ≥ 3 months)	3 (8.6%)	2 (12.5%)	
	Routine primary IOL implantation when capsular support adequate	30 (85.7%)	11 (68.8%)	
Preferred IOL material	Heparin-surface-modified (HSM) PMMA	13 (37.1%)	7 (43.8%)	0.270
	Hydrophilic acrylic	6 (17.1%)	0 (0.0%)	
	Hydrophobic acrylic	16 (45.7%)	9 (56.3%)	
Preferred intraoperative anti-inflammatory adjunct	Intracameral steroid (Prednisolone/Dexamethasone)	7 (20.0%)	4 (25.0%)	0.943
	Intravitreal Triamcinolone	1 (2.9%)	0 (0.0%)	
	No adjunct (standard postoperative regimen only)	4 (11.4%)	2 (12.5%)	
	Posterior Sub-Tenon’s Triamcinolone	7 (20.0%)	4 (25.0%)	
	Subconjunctival steroid (Prednisolone/Dexamethasone)	16 (45.7%)	6 (37.5%)	
Postoperative systemic corticosteroid strategy	Avoid routine use; reserved for inflammatory surge	7 (20.0%)	5 (31.3%)	0.376
	Routine use in all uveitic cataract patients	24 (68.6%)	8 (50.0%)	
	Routine use in high-risk cases only	4 (11.4%)	3 (18.8%)	
Duration of postoperative topical corticosteroid therapy	3 months	3 (8.6%)	0 (0.0%)	0.612
	4 weeks	4 (11.4%)	3 (18.8%)	
	6–8 weeks	8 (22.9%)	2 (12.5%)	
	Tailored to inflammation response and uveitis type	20 (57.1%)	11 (68.8%)	
Approach to use of post-op topical NSAIDs for CME prophylaxis?	Avoid; rely on topical corticosteroids alone for prophylaxis	5 (14.3%)	1 (6.3%)	0.711
	Initiate only if CME is detected postoperatively (OCT/angiography)	12 (34.3%)	4 (25.0%)	
	Prescribe only for high-risk cases (e.g., posterior uveitis, prior CME)	3 (8.6%)	2 (12.5%)	
	Routine use in all cases	15 (42.9%)	9 (56.3%)	
First-line intervention for inflammation refractory to maximal topical therapy	Sub-Tenon’s steroid injection	15 (42.9%)	4 (25.0%)	0.385
	Initiate/augment systemic corticosteroids	14 (40.0%)	7 (43.8%)	
	Intensify topical steroid frequency	1 (2.9%)	0 (0.0%)	
	Investigate for infection or retained lens material	5 (14.3%)	5 (31.3%)	
Support for national standardized management guidelines for uveitic cataract surgery?	Cautiously support; dependent on data confidentiality and utility	5 (14.3%)	3 (18.8%)	0.694
	Strongly support; essential for standardizing care	30 (85.7%)	13 (81.3%)	

N: Frequency, %: Percentage, *p < 0.05, significant, Chi-square test, Fischer Exact test

## Discussion

This is the first comprehensive survey to evaluate perioperative management practices for uveitic cataract among ophthalmologists in Pakistan, highlighting areas of agreement as well as opportunities for greater standardization. This study describes existing practice patterns rather than establishing clinical recommendations; findings are intended to inform, not substitute for, future evidence-based consensus guidelines. There was considerable variation in practices among the participants as had been reported in the international community. However, a vast majority (84.3%) of our participants agreed to the need for formulation of the national guidelines to improve outcomes. The survey achieved a 51% response rate, and while respondents represented a range of clinical backgrounds, the findings are best interpreted as reflective of practice patterns among network-accessible, largely tertiary-care-affiliated ophthalmologists in Pakistan rather than the ophthalmology community as a whole. Differences in clinical practices were observed, which may reflect variations in available resources and clinical settings.

Much of the historical framework for uveitic cataract management, including early guidance from the AAO Preferred Practice Patterns, predates 2012 and offers limited procedural specificity for this population. More recent evidence, however, has better characterized real-world variation and outcomes: a 2017 UK multicentre database study highlighted inconsistent complication rates across centers despite established protocols [8], and updated SUN Working Group criteria (2022) have since refined disease-activity classification relevant to surgical timing [9]. Regionally, recent Pakistani data on uveitis etiology and outcomes (2019, 2025) further underscore how infectious causes and resource variability, largely unaddressed in older Western benchmarks, shape real-world decision-making [11, 12]. Within the scope of our sample, our findings contribute empirical, practice-level data from a resource-varied South Asian setting, addressing a gap that persists even in recent global literature: limited insight into how perioperative protocols are actually applied outside high-resource, single-center settings.

According to our survey, 72.5% of experts recommended surgery based on a fixed duration of quiescence (e.g., ≥ 3 months without inflammation), making it the primary factor influencing surgical timing whereas 35.5% prioritize the degree of inflammatory control achieved with the current treatment regimen. These findings align with established guidelines and practical approaches, showing a strong consensus among experts. International Uveitis Study Group (IUSG) survey also showed 3 months preferred quiescence period by 70% of responders [13]. Quiescent inflammatory period of at least 3 months prior to cataract surgery is associated with a reduced rate of complications in postoperative period especially CME [14]. However, the etiology of uveitis also plays an important role in this decision making. In childhood uveitis and Behcet ‘s uveitis, 6 months period of disease inactivity is recommended preoperatively whereas in Vogt Koyanagi Harada disease, one month quiescence is sufficient [15, 16].

Regarding the intensification of topical corticosteroids, varied practices were shown. Majority of respondents (33.3%) adopted a reactive approach, increasing therapy only if active inflammation is present preoperatively. A significant proportion (31.4%) preferred intensification 1 week before surgery, indicating a prophylactic approach aimed at pre‑empting postoperative inflammation which was comparable to study conducted by Mora et al.^17^ Smaller groups opted for intensification 1–3 days (19.6%) or 2–4 weeks (15.7%) pre‑operatively, reflecting differing views on the optimal timing for corticosteroid augmentation [17–19].

These findings highlight a lack of consensus on the optimal timing and duration of topical steroids in uveitic cataract cases, with clinicians balancing the risk of exacerbating inflammation against the potential side effects of prolonged steroid exposure [20].

Managing infectious uveitis can be challenging, as it requires balancing infection control with the need to address cataract-related visual impairment. The majority (56.9%) approach of deferring surgery until quiescence was achieved without antimicrobial coverage aligns with consensus recommendations, though the substantial minority intensifying coverage (15.7%) or continuing existing antimicrobial therapy uninterrupted through surgery (13.7%) reflects appropriate clinical judgment in higher-risk scenarios and showed consistency with results reported by Chatterjee et al. [21]. Given the higher burden of tuberculous uveitis and other infectious aetiologies in the Pakistani context compared to Western populations, clearer guidance on perioperative antimicrobial management would be particularly valuable for local practice.

Managing patients on systemic immunosuppression or biologic therapy demands meticulous planning. Biologics are instrumental in achieving state of quiescence and in management of refractory uveitis [22]. Our survey results indicated that most experts (39.2%) customised perioperative management based on the specific drug, dose, and inflammation status, highlighting a tailored approach to balance disease control and surgical risk. A substantial proportion (33.3%) preferred continuing the current regimen without modification, suggesting confidence in the existing therapeutic control and showing consistency with the Turkish expert survey which continues biologic therapy at its current dose through the preoperative period to prevent inflammatory flares [23]. Meanwhile, 21.6% chose to augment therapy with perioperative systemic steroid cover to prevent inflammation spikes. Only a few deferred decisions to rheumatologists (3.9%) or withhold biologics (2.0%), indicating these strategies are less favoured. Our findings underscore the importance of personalized risk assessment and inflammation control in optimizing surgical outcomes for uveitic cataract patients.

The preference for the dominant strategy for managing intraoperative miosis or posterior synechiae was a combined pharmacologic‑mechanical approach (74.5%) in our survey, acknowledging that uveitic eyes often have pupillary abnormalities. Fewer surgeons used isolated methods i.e. mechanical synechiolysis (7.8%), pharmacologic visco‑synechiolysis (3.9%), or pupil‑expansion devices (13.7%) [14, 24]. This suggests focus should be on refining the combined technique could be an area worth exploring in future guideline development to improve intraoperative pupil management.

The survey results indicate that most surgeons (80.4%) adopted a routine primary intraocular lens (IOL) implantation strategy in uveitic cataract cases, provided capsular support was adequate. This reflects an evolving confidence in managing uveitic inflammation intraoperatively and postoperatively, enabling immediate visual rehabilitation while 9.8% restricted it to special cases and another 9.8% insisted on a preoperative inflammatory quiescence emphasizing the importance of inflammation control prior to IOL implantation [25].

The strong preference for hydrophobic acrylic IOLs (49.0%) was consistent with evidence showing they elicit a reduced inflammatory response and exhibit good biocompatibility compared to hydrophobic acrylic and Polymethyl Methacrylate (PMMA) materials in uveitic eyes. Heparin‑surface‑modified (HSM) PMMA lenses were selected by 39.2% of respondents, valued for their anti‑adhesive surface. Hydrophilic acrylic lenses were used by 11.8%, though contemporary evidence increasingly favours hydrophobic acrylic as the material of choice [26, 27]. The discussion highlights that material selection should be tailored based on the patient’s uveitis severity and propensity for postoperative inflammation. Further studies are needed to assess long‑term material‑related outcomes in uveitic populations.

Intraoperative anti‑inflammatory adjuncts in uveitic cataract surgery are necessary to prevent postoperative inflammation, fibrin formation in anterior chamber and cystoid macular edema [14, 28]. In our survey, 43.1% of respondents preferred subconjunctival steroid (Prednisolone/Dexamethasone) to achieve localized, sustained inflammation control with minimal systemic effects. Intracameral steroid and posterior sub‑tenon’s triamcinolone were each used by 21.6% of respondents, offering direct intraocular or periocular anti‑inflammatory action. Only 2.0% chose intravitreal triamcinolone. Our results were in contrast with study by Caglar et al. which demonstrated more effectiveness of intracameral steroids than subconjunctival steroids in controlling inflammation [29].The choice of adjunct should weigh effectiveness against risks like elevated intraocular pressure or infection. Subconjunctival injection is favored for its simplicity and local efficacy, while intracameral or sub‑tenon’s delivery is useful for targeted anterior segment control. Additional research is needed to compare long‑term outcomes of these adjuncts and optimize management protocols in uveitic cataract surgery.

Postoperatively, most surgeons (62.7%) opted to give systemic corticosteroids routinely to all uveitic cataract patients, while 23.5% used them only for post‑op inflammation surges and 13.7% reserved them for high‑risk cases. Our results show consistency with Multicenter Uveitis Steroid Treatment (MUST) Trial which indicated uveitic eyes with cataract treated with standard systemic steroid therapy had better visual outcomes [30].

However, tapering protocol did not show consistency with the available literature [31]. 60.8% respondents tailored the treatment duration to the patient’s inflammation response and uveitis type; fewer used fixed periods (6–8 weeks, 4 weeks, or 3 months). This approach combines routine steroid prophylaxis with tailored strategies, weighing individual risk factors to manage inflammation while reducing side effects. Our survey also revealed that clinicians most often choose to initiate or augment systemic corticosteroid therapy (41.2%) as the first intervention in inflammatory eyes refractory to maximal topical steroids, followed by sub-tenon’s steroid injection (37.3%). Customizing treatment is key for better outcomes with minimal complications in uveitic cataract surgery [32, 33].

For postoperative CME prevention, topical NSAIDs drops play an essential role. Combining them with steroids appear to be more effective as shown by studies [34]. 47.1% surgeons responded that they routinely use topical NSAIDs for CME prophylaxis which aligns with established efficacy of NSAIDs in preventing CME. The risk stratified approach adopted by 9.8% of respondents is reasonable. Surgeons with ≤ 25 uveitic cataract cases per year were more likely to avoid NSAIDs (14.3%) than high‑volume surgeons (> 25 cases, 6.3%). Factors such as cost and drug availability likely influenced the prescribing patterns. The high uptake of postoperative OCT surveillance (31.4%) reflected appropriate prioritization of CME detection, a key determinant of visual outcomes. Efforts to improve access to OCT technology or develop alternative surveillance strategies in resource limited settings are warranted [35, 36].

However, regarding aetiology in cases of postoperative CME with a quiet anterior chamber, the majority suspected persistent subclinical uveitis (56.9%) as the primary aetiology, highlighting the importance of controlling low‑grade inflammation which is consistent with results shown in recent literature [37]. Breakdown of the blood‑aqueous barrier (29.4%) was the next most opted cause.

As far as postoperative mydriatic‑cycloplegic strategy was concerned, 45.1% prescribed only for inflammation or synechiae risk, and 43.1% gave it for 1–2 weeks in all cases. The risk‑based approach is supported by evidence that targets cycloplegics to high‑risk eyes (e.g. to prevent postoperative posterior synechiae, persistent iritis) while routine use may be unnecessary in low‑risk patients [38, 39].

For follow‑up, 33.3% respondents preferred an intensive schedule, 19.6% followed a standard cataract protocol, and 47.1% tailored visits to individual disease activity. The individualized schedule matches guidelines that recommend personalized monitoring for uveitic eyes to better detect CME and manage inflammation [2, 40].

Regarding prediction of visual outcomes after uveitic cataract surgery, 45.1% respondents considered preoperative macular integrity on OCT as the strongest single predictor of visual gain, highlighting the importance of OCT and imaging techniques. The anatomical type of uveitis and the duration of preoperative quiescence each received 25.5% of responses, suggesting these factors were considered important but secondary to macular status. Study by Jevnikar et al. supported these findings, demonstrating that OCT-based assessment of macular structure, such as foveal thickness and ellipsoid zone integrity, is strongly associated with final visual acuity in patients with uveitic cataract [41, 42].

Our survey highlights both similarities and differences in national practice patterns, as well as variations when compared with international practices.

Differences in uveitic cataract management preferences according to surgeon experience and annual case volume suggest potential gaps in standardization and training. Young consultants with experience (≤ 10 years) tend to rely more on intensified topical corticosteroids preoperatively, possibly reflecting a more cautious approach. In contrast, more experienced surgeons (> 10 years) favor systemic steroids and prophylactic NSAIDs, suggesting a more aggressive management strategy based on experience. Higher-volume surgeons (> 25 cases/year) emphasized a fixed quiescence period more (81.3%) than lowervolume surgeons (≤ 25 cases/year). These differences indicate that management strategies are influenced by surgeon’s experience and expertise. Standardizing care pathways while accommodating surgeon expertise could help refining patient care and improving outcomes. The preference for hydrophobic acrylic IOLs, routinely implanting IOL in adequate capsular support aligns with surveys from the US, UK, and Europe, indicating global consensus [2, 18]. However, Pakistani practitioners more often opted for perioperatively subconjunctival steroids (43.1%), diverging from international trends favoring intracameral and intravitreal steroids [29]. The use of preoperative steroid also varied, and no single protocol was consistently followed. These differences may reflect variations in patient characteristics, access to healthcare resources, or individual clinician preferences. The strong support for national guideline development (84.3%) highlights the need for context-specific recommendations to help reduce practice variation and improve surgical outcomes.

### Strengths of study

This study has several strengths that support the reliability and relevance of its findings. To our knowledge, it is the first systematic assessment of perioperative management practices for uveitic cataract in Pakistan, providing a useful baseline for future research and guideline development. The questionnaire was carefully designed and reviewed to ensure that it captured clinically relevant information. In addition, the respondents represented a range of clinical backgrounds and practice settings, which supports the relevance of the findings to similar tertiary-care-oriented practice settings in Pakistan, though not necessarily to the full national ophthalmology community. The study followed established reporting practices to maintain transparency, and the inclusion of the survey instrument may assist future research in this area. By examining multiple aspects of perioperative care, the study offers a comprehensive overview of current practice patterns and provides insights that may help inform clinical decision-making.

### Study limitations

Our survey has several limitations that should be considered when interpreting the findings. The survey design limited deeper insight into the reasoning behind clinicians’ management strategies, and the relatively small sample size (*n* = 51) may restrict generalizability. As no centralized registry of uveitis-specialized ophthalmologists exists in Pakistan, the true size of the eligible national population could not be precisely determined, limiting our ability to formally assess sample representativeness. Institutional context was also not analyzed as a separate variable. In addition, responses may reflect reported preferences rather than actual clinical practice, and the study focused on management approaches rather than clinical outcomes. Selection bias is also possible, as those who responded may differ from those who did not, and the sample may overrepresent academic practitioners while underrepresenting those working in private or remote settings. Non-response bias is a related concern: of 100 consultants invited, 51 responded, and those who took the time to complete the survey may be more engaged or protocol-driven than those who didn’t, which could make reported practices look somewhat more standardized than they actually are nationally. Geographically, most respondents were likely reached through hospitals and networks centred in major cities, so practice patterns in smaller towns or under-resourced areas may not be well captured. Finally, the cross-sectional design captures practices at a single point in time and may not reflect evolving trends. These limitations suggest that the findings should be interpreted with caution, and further research is needed to better understand how these practices relate to patient outcomes.

## Conclusion

This nationwide survey demonstrates considerable variation in the perioperative management of uveitic cataract among ophthalmologists in Pakistan, identifying specific, actionable targets for standardization. These findings represent an observational baseline rather than clinical guidance and are intended to inform, not replace, future consensus-based guideline development. While most clinicians preferred a minimum of three months of inflammatory quiescence before surgery, approaches to preoperative steroid intensification varied. Hydrophobic acrylic intraocular lenses were the most commonly preferred, reflecting alignment with current evidence. Subconjunctival corticosteroid injections were frequently used intraoperatively, although practices differed regarding localized versus systemic anti-inflammatory prophylaxis. Preoperative macular integrity on OCT was widely regarded as an important predictor of postoperative visual outcomes. Postoperative management was often individualized, which may make comparison of outcomes across centers more challenging. Differences in management approaches were also observed between less experienced and more experienced surgeons. Importantly, a large majority of respondents supported the development of national standardized guidelines, highlighting the need for context-specific recommendations to improve consistency of care and surgical outcomes. These findings offer local healthcare systems and clinical policymakers concrete starting points, including corticosteroid protocols, standardized postoperative follow-up, and experience-based training support, for developing such guidelines.

## Data Availability

The datasets generated and/or analyzed during the current study, along with the survey instrument, are available from the corresponding author on reasonable request.

## Abbreviations

AAO: American Academy of Ophthalmology
CME: Cystoid Macular Edema
HSM: Heparin-Surface-Modified
IOL: Intraocular Lens
IUSG: International Uveitis Study Group
NSAID: Non-Steroidal Anti-Inflammatory Drug
OCT: Optical Coherence Tomography
PMMA: Polymethyl Methacrylate
SUN: Standardization of Uveitis Nomenclature
